# Humans and Ecosystems Over the Coming Millennia: Overview of a Biosphere Assessment of Radioactive Waste Disposal in Sweden

**DOI:** 10.1007/s13280-013-0405-7

**Published:** 2013-04-26

**Authors:** Ulrik Kautsky, Tobias Lindborg, Jack Valentin

**Affiliations:** 1 Swedish Nuclear Fuel and Waste Management Co. (SKB), Box 250, 101 24 Stockholm, Sweden; 2 Öregrundsgatan 15, 115 59 Stockholm, Sweden

**Keywords:** Hazard assessment, Radionuclides, Dose, Greenhouse warming, Forecast

## Abstract

This is an overview of the strategy used to describe the effects of a potential release from a radioactive waste repository on human exposure and future environments. It introduces a special issue of *AMBIO*, in which 13 articles show ways of understanding and characterizing the future. The study relies mainly on research performed in the context of a recent safety report concerning a repository for spent nuclear fuel in Sweden (the so-called SR-Site project). The development of a good understanding of on-site processes and acquisition of site-specific data facilitated the development of new approaches for assessment of surface ecosystems. A systematic and scientifically coherent methodology utilizes the understanding of the current spatial and temporal dynamics as an analog for future conditions. We conclude that future ecosystem can be inferred from a few variables and that this multidisciplinary approach is relevant in a much wider context than radioactive waste.

## Introduction

**Fig. 1:**
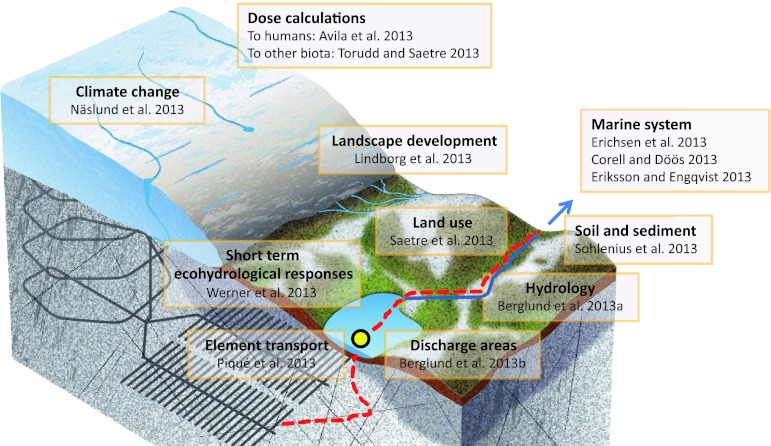
Conceptualization of the radionuclide flow path from a repository and surface ecosystems exemplified with periglacial conditions. The articles of the present *AMBIO* special issue (vol. 42:4, 2013) are displayed to show their context in the assessment. *Red dashed line* indicates hypothetical flow path of radionuclide. *Blue arrow* links the terrestrial and limnic ecosystems with marine basins. *Yellow dot* exemplifies discharge area of deep ground water. *Gray shaded areas* shows permafrost and the repository is outlined with *black lines* in *gray bedrock*

Any risk assessment dealing with long-term safety must understand and characterize those aspects of the environment that can be critical in the future. For long-lived radioactive waste, the time frame comprises at least some thousands of years and can extend up to about 1 million years. In this article, we look into the crystal ball of the coming millennia, by summarizing 13 articles in this special issue of *AMBIO*. Different methods for identifying and characterizing possible futures that would affect the potential risks of a nuclear waste repository are discussed and evaluated (Fig. 1).

The major parts of the research and analysis were performed within the so-called SR-Site project, a Safety Report for the assessment of a repository for spent nuclear fuel at the Forsmark Site. The results come from a decade of multidisciplinary research aiming to understand how radionuclides from a potential release from such a repository might migrate and cause radiation exposures to humans and the environment in the far future. The underlying data have been reported in a series of reports (see SKB 2011 for an overview). The special issue highlights results that we believe will be of general interest to a wider audience. The articles focus on analyses that were performed relating to the surface ecosystems, that is, the biosphere. Associated analyses for other disciplines are summarized in other articles or special issues, for example, hydrogeology (Selroos and Painter 2012; Selroos et al. 2012) and geochemistry (Gascoyne and Laaksoharju 2008).

We hope that this special issue will stimulate discussions on how to make projections of the future, and that the wealth of underlying data will encourage further analyses or alternative interpretations.

### The Safety Assessment, SR-Site

**Fig. 2:**
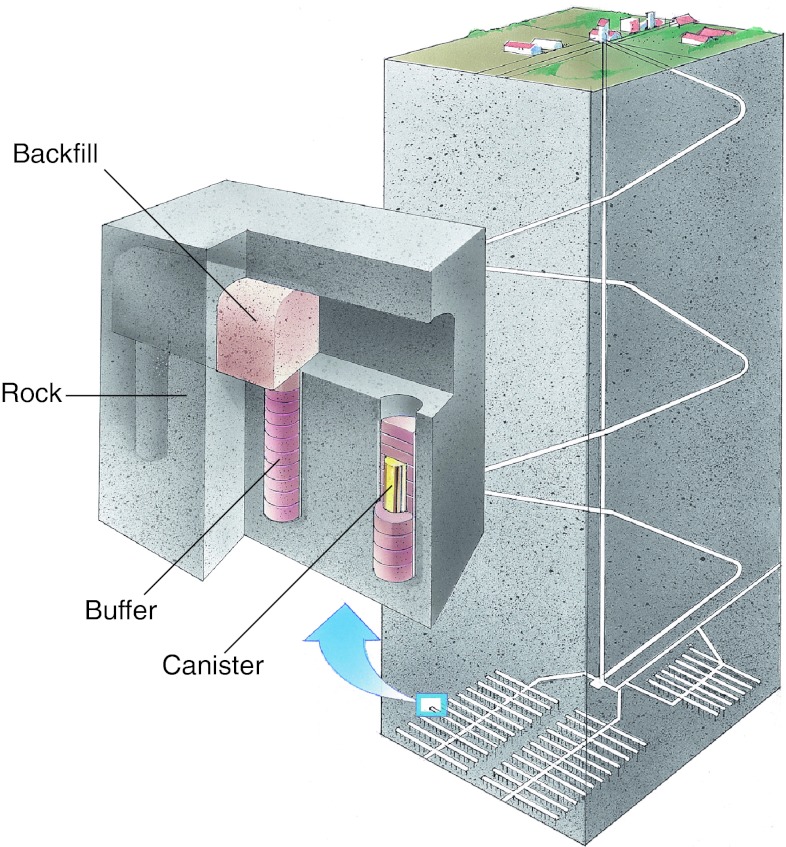
The KBS-3 method. Copper canisters with a cast iron insert containing spent nuclear fuel are surrounded by compacted bentonite clay and deposited at approximately 500 m depth in groundwater saturated, granitic rock

**Fig. 3:**
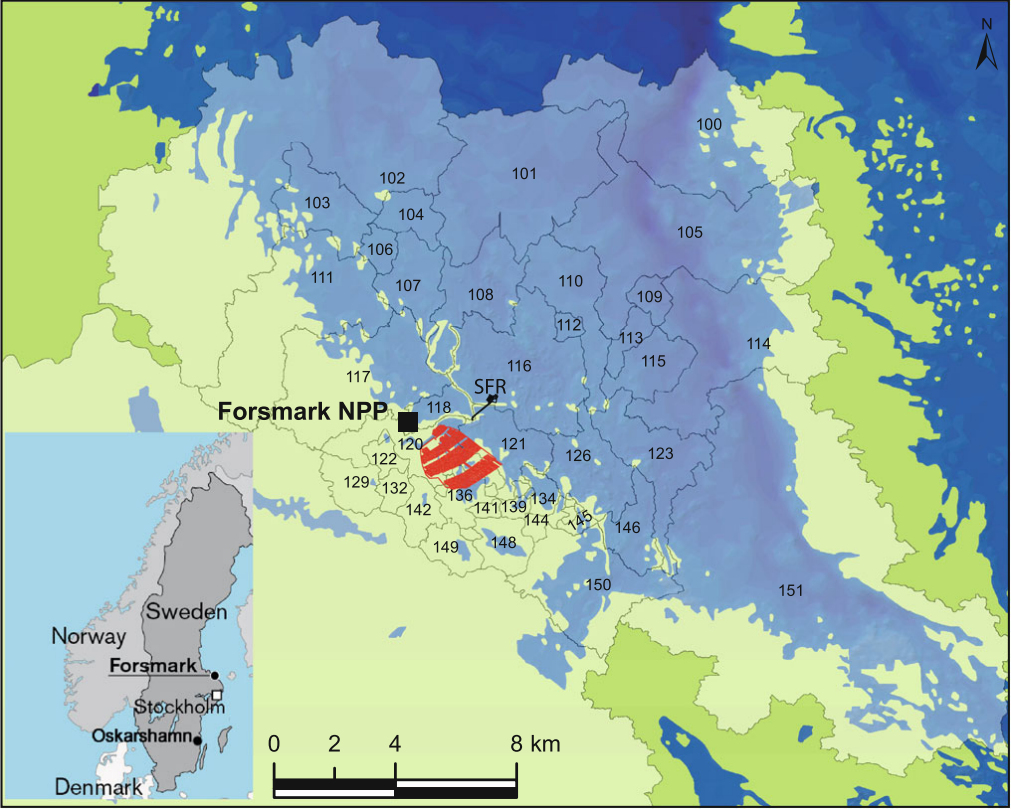
Location of the Forsmark and Oskarshamn sites in Sweden (*inset*) and map of Forsmark area with the shoreline of the Baltic Sea. *NPP* Nuclear power plant; *SFR* low level waste repository; *numbers* denote “basins” used in the modeling (Lindborg et al. 2013). *Red hatched area* shows planned extent of a tentative repository for spent nuclear fuel

The purpose of the safety assessment project SR-Site (SKB 2011) was to investigate whether a safe spent nuclear fuel repository of the so-called KBS-3 type (Fig. 2) can be built at the Forsmark site, situated in the municipality of Östhammar, Sweden. The Forsmark site (Fig. 3) was selected based on findings emerging from several years of site investigations at Forsmark and also at the Laxemar-Simpevarp site in the municipality of Oskarshamn (Andersson et al. 2013). Earlier results from this study have been published in a previous special issue of *AMBIO* (Lindborg et al. 2006).

The report of the SR-Site project (SKB 2011) is a main component in the application of SKB (Swedish Nuclear Fuel and Waste Management Co.) for a license to build a KBS-3 repository at Forsmark. The role of the SR-Site report in the application is to demonstrate long-term safety after closure of the repository. That report, and all background reports that it summarizes, are downloadable from the SKB web site, http://www.skb.se/publications.

In the KBS-3 method, copper canisters with a cast iron insert containing spent nuclear fuel are surrounded by compacted bentonite clay and deposited at approximately 500 m depth in groundwater saturated, granitic rock, see Fig. 2. The purpose is to isolate the nuclear waste from humans and the environment for very long times. Around 6000 canisters containing a total of 12 000 tons of spent nuclear fuel are forecast to arise from the current Swedish nuclear power program.

According to the authorities, a main acceptance criterion for a repository is that the annual risk of harmful effects after closure does not exceed one per one million for a representative individual of the most exposed group (SSM 2008). The risk limit corresponds to an annual effective dose to humans of about 1.4 × 10^−5^ Sv (sievert), i.e., in the order of one percent of the effective dose due to natural background radiation in Sweden (UNSCEAR 2008). The time scale of a safety assessment for a final repository for spent nuclear fuel is specified in the regulations to be 1 million years after closure and a detailed risk analysis is required for the first 1000 years after closure. For the period up to approximately 100 000 years, conclusions are to be based on a quantitative risk analysis (SSM 2008).

Over such extended periods of time, it is obvious that the evolution of a repository can never be fully described or understood. Thus, a central theme in any safety assessment methodology is the management of all relevant types of uncertainties. That means identifying, classifying, and describing uncertainties, as well as handling them in a consistent manner in the quantification of the evolution of the repository and of its radiological consequences. The safety assessment, SR-Site, consists of 11 main steps (SKB 2011). Briefly, the first steps comprise identifying FEPs (features, events, and processes) influencing the disposal system, characterizing its initial state and describing the external conditions that affect it. In subsequent steps, safety functions of the repository are defined and a reference evolution of the repository system is analyzed, based on the assumption that the last 120 000-year glacial cycle is repeated. Based on the results of the reference evolution and of the safety functions of the repository, a number of scenarios are defined. These aims at exploring whether the integrity of the waste canisters can under any circumstances be jeopardized, systematically taking all identified uncertainties in the evolution of the repository into account. In the scenarios leading to releases of radionuclides from the canisters, transport of radionuclides from the repository to the surface environment and the resulting risks to humans and the environment are estimated. This is finally evaluated and discussed with respect to compliance with the regulatory risk criterion.

**Fig. 4:**
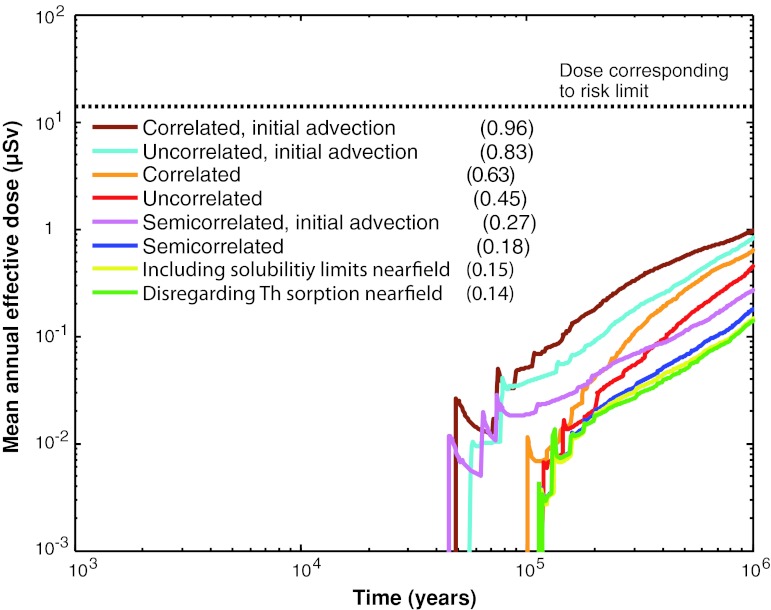
Summary of mean annual effective dose for all probabilistic calculations performed for the corrosion scenario, the main contributor to risk in the SR-Site assessment. The peak doses are given in *parentheses* in μSv. In the legend, “Correlated,” “Uncorrelated,” and “Semicorrelated” refer to three variants of the hydrogeological model used in SR-Site. The three “initial advection” cases put upper bounds on the possible consequences of buffer degradation processes (SKB 2011)

The main conclusion of the safety assessment SR-Site is that a KBS-3 repository that fulfils long-term safety requirements can be built at the Forsmark site. The detailed analyses demonstrate that canister failures in a 1-million year perspective are rare and even with a number of pessimistic assumptions the modeled radiological consequences are well below one percent of the natural background radiation (Fig. 4).

In the SR-Site project climate models and assumptions related to future climate states are presented in detail in SKB (2010a) as recapitulated in this issue of *AMBIO* by Näslund et al. (2013), while the characteristics of surface ecosystems are compiled in a synthesis document (SKB 2010b). That document summarizes nearly 20 reports concerning the biosphere component of SR-Site. It covers the philosophy of the biosphere assessment, which is to make estimates of the radiological risks to humans and the environment that are as relevant as possible, based on the knowledge of present-day conditions at Forsmark and the past and expected future development of the site. It contains brief summaries of the extensive ecosystem studies, including oceanographic and hydrological studies used in the special issue.

This interdisciplinary and synchronous large-scale collection and analysis of site data makes Forsmark one of the most extensively investigated sites used in risk or environmental impact assessments. By combining the expected discharge of deep groundwater from the repository location with landscape geometries, “biosphere objects” (that is, the areas most likely to be affected by a potential release of radionuclides) were identified and outlined (cf. Lindborg 2010). The properties and temporal development of these discharge areas were described as they developed from coastal sea basins to terrestrial ecosystems. The identified biosphere objects represent considerable variations in size, timing of the start of succession, and object-specific properties.

## The Site

Details concerning the site can be found in Lindborg (2008). The area represents a typical coastal site at the shoreline of the Baltic Sea in northern Uppland, Sweden (Fig. 3). The latest deglaciation in Forsmark took place c. 10 800 years ago and the area was then covered by c. 150 m of water. Glacial deposits, mainly till with a rich content of calcite, originating from the sedimentary bedrock of Gävlebukten about 100 km north of Forsmark, were deposited on the granitic bedrock. The closest shore/land area at that time was situated c. 80 km to the west of Forsmark. The rapid shoreline displacement has affected landscape development strongly, and still causes a continuous and relatively predictable change in the abiotic and biotic environments. The first parts of Forsmark emerged from the sea around 500 bc.

Post-glacial land uplift, in combination with the flat topography (altitudes less than 20 m), implies fast shoreline displacement that has resulted in a young terrestrial system that contains a number of newborn shallow lakes and wetlands. The lakes themselves are also of a unique oligotrophic hardwater type that is only found in northern Uppland. The lakes are small (<0.6 km^2^) and shallow (0.6–2 m deep) and have both a high pH and high concentrations of major constituents (e.g., calcium and bicarbonate), as well as a water turnover time of less than 1 year. Occasionally, the low lying lakes are flooded by the sea during periods with high sea levels.

The coast is open toward north-east and subjected to high wave erosion and rapid water turnover of some days. This gives a seabed that is dominated by erosion and transport bottoms consisting mainly of sand and gravel with varying fractions of glacial clay, and close to the mainland rocky bottoms and coarse till.

The terrestrial vegetation is affected by the nature of the regolith and human land use. Thus, conifer forests are common on the dominant wave-washed till. The calcareous influence is manifested in the under-vegetation with a rich flora. Wetlands occur frequently and cover 10–35% of the area in the three major catchments. A major part of the wetlands are coniferous forest swamps and open mires. Arable land, pastures, and clear-cuts dominate the open land. Arable land and pastures are found close to settlements. The pastures have been intensively used, but today are a part of the abandoned farmland.

The annual precipitation and runoff are 560 and 150 mm, respectively. No major water courses flow through the central part of the site and small brooks can be dry for long periods during dry years.

## The Biosphere Assessment

This biosphere assessment has several novel components. First of all, substantial site information is available from a site investigating program designed to improve site-specific data and understanding of the biosphere at Forsmark and the alternative candidate site, Laxemar-Simpevarp (Lindborg et al. 2006). The program generated one of the largest databases currently available in Sweden on simultaneously measured ecosystem data covering marine, limnic, and terrestrial ecosystems (Andersson 2010; Aquilonius 2010; Löfgren 2010; Tröjbom and Nordén 2010; Bradshaw et al. 2012). The database also includes information on, for example, sorption in soils and transfers to organisms for about 40 elements, which is a substantial addition to other available data sources such as IAEA (2010).

The high spatial resolution (the digital elevation model, DEM) and temporal inter-annual resolution of the database together with historical data provides a good understanding of the landscape changes (Lindborg et al. 2013).

An ecosystem approach has been used to understand the major fluxes and pools of water and organic matter in the landscape and estimate the constraints on the systems (e.g., water resources, available area, primary production, and import/export; Andersson and Sobek 2006; Jansson et al. 2006; Kumblad et al. 2006; Löfgren et al. 2006; Sobek et al. 2006; Wijnbladh et al. 2006; Saetre et al. 2013).

This rich information provides a unique opportunity to explore the future to an unusual extent in safety assessments. The future can be envisaged by looking at older parts of the site, as almost all development stages of the landscape can be found within the investigation area. Thus, today’s biosphere could function as a natural analog of the future landscape (Lindborg et al. 2013). The ecosystem approach maintains mass balances by systematic book-keeping of matter and fluxes (Kumblad et al. 2006; Löfgren et al. 2006). This sets constraints on how humans can utilize the landscape (e.g., Jansson et al. 2006), which is primarily driven by the availability of water, area, and the productivity of different food sources (Saetre et al. 2013).

The fluxes of radionuclides and consequent exposures to humans and the environment are associated with the fluxes of matter. The sizes of present and future lake sub-catchment areas set constraints on how much water is available to transport radionuclides to a specific area. Lake basins and other low land areas set the limits for areas affected by deep groundwater discharges (Berglund et al. 2013b). These areas can also accumulate radionuclides and have subsequent potential for agriculture activities and are defined as biosphere objects for assessment. Thus, the future is presented as a reasonable and consistent derivation from the present conditions at the site, rather than as a collection of narratives or scenarios.

The context of the articles in this special issue is illustrated in Fig. 1 and further discussed below.

## Various Way to Handle the Uncertainty of the Future

The understanding of all aspects of future development for a specific site will always be vitiated with a degree of uncertainty. In the following, some examples are given of how to handle the uncertain future. The examples are not predictions but a set of reasonable speculations of different alternative futures, especially those that can be relevant for calculating radiation doses to future humans and the environment.

A common initial approach to handle the future is to assume that it will not be significantly different from present conditions. Some assumptions to be made about the future that are needed in dose calculations are regulated by legislation and defined as constants, for example, the dose conversion factors relating radiation dose to intake of activity (summarized most recently in ICRP 2012). Thus, for regulatory purposes human physiology and the ability to treat cancers are assumed to remain constant. There have been several attempts to define a “reference biosphere,” in its simplest form as one biosphere valid for all times and sites (van Dorp et al. 1998). Usually, this comprises a drilled well, assuming that the well is the main mediator of contaminated water transfer to the biosphere. However, the approach has been subject to criticism and it is obvious that the range of potential types of well will give large variations in the assessed radiation dose; also, other recipient environmental media give higher doses for some radionuclides (Avila et al. 2010).

If the perspective is widened, random variation of parameter values will be a representation of all kinds of biospheres, where the parameter values are selected from distributions in a Monte Carlo simulation. Usually, this variation is described using probability density functions (PDFs) based on measurements of the parameters. This does not necessarily require a full understanding of what causes the variations, but correlations between parameter values can be used to reduce unlikely combinations of values. This methodology has been widely used in biosphere assessments during the last three decades and indeed in several studies in this special issue (Avila et al. 2013; Erichsen et al. 2013). Erichsen et al. (2013) compare two marine ecosystem models, one with high temporal and spatial resolution, which gives variations between marine basins over the year. The other model has a coarser spatial and temporal resolution, but uses as input random variations from Monte Carlo simulations. Both models give similar results for concentration ratios (CR) for fish, which are comparable with measured site data (Bradshaw et al. 2012). The disadvantages with the high-resolution model are the large requirement for data and the protracted computation time, but the gain in mechanistic understanding that is developed using this model shows that bio-concentration factors for point sources vary over time and space contradicting the assumptions often applied when using this type of factor.

Avila et al. (2013) present work based on a methodology using Bayesian inference to handle PDFs for small and skewed distributions and address several ways of estimating uncertainties in the dose calculations for humans. Bayesian inference is used to combine the few representative controlled site data with a larger number of heterogeneous data of unknown precision from the entire world (Nordén et al. 2010; Avila et al. 2013).

Beside temporal and spatial variations (Lindborg et al. 2013), concentration factors (CR) and sorption factors (*K*_D_) are the most important determinants of dose to humans from radionuclides. For many elements, *K*_D_ and CR values are available from compilations by IAEA (2010). The numbers of observations, methods, and quality of observations differ between studies and between data obtained from various parts of the world. Thus, PDFs based on these studies can have long tails reflecting the low number of observations and variations due to the use of data from various sites obtained by different methods. In contrast, for many elements, the site studies in the SKB program (Lindborg 2006) have produced a limited range of CR and *K*_D_ values using consistent methods, as shown by Sohlenius et al. (2013).

Näslund et al. (2013) describe how the variation in climate and related processes such as formation of ice sheets, growth of permafrost, and changes in sea level are understood and generalized for the safety assessment. They show that, for the deep repository for spent nuclear fuel, the climate scenarios range from cases with high-end global warming for the coming 100 000 years, through to cases with maximum deep permafrost, to cases with large ice sheets during full glacial conditions. The safety assessment for the planned KBS-3 repository for spent nuclear fuel extends to 1 million years, over which period about eight glacial–interglacial cycles are expected.

The large-scale climate variations affect the shore line displacement and the ingrowth of lakes. Shore line displacement is, at present, about 0.3 m in 50 years at Forsmark, thus sufficient to be observed by inhabitants at the coast. Therefore, it is obvious that climate change and shore line displacement need to be addressed in a safety assessment, either through calculations where these processes are included, or at least through a discussion of those aspects that it is not necessary to represent explicitly in assessment calculations.

The past shore level displacement is measureable (Påsse 2001; Påsse and Andersson 2005), over a short time frame it can be extrapolated into the future (Brydsten and Strömgren 2010), and for longer time frames it can be modeled (Näslund et al. 2013). Shore line displacement affects the geometry, i.e., bathymetry or topography, which determines the size and shape of the emerging land and initial formation of lakes, see Lindborg et al. (2013). Geometry is measurable with good precision for both the land and the sea and is assumed to be relatively constant over time except for the sedimentation and in-growth of vegetation is described by Lindborg et al. (2013).

Thus, only one time varying parameter, shore line displacement, together with one almost time-independent parameter, geometry, gives a complex site-specific variation of the landscape over time.

The climate-induced changes in land formation also affect other processes through the geometry of the environment. Besides radioactive decay, the most important processes for the assessment are dispersal and accumulation of radionuclides, and exposure to those radionuclides, which affect the risks to both the humans and the environment. The dispersal of radionuclides from the underground repository is predominantly by water which transports and dilutes the radionuclides, while accumulation processes comprise sorption, precipitation, and uptake in organisms.

In this special issue, Eriksson and Engqvist (2013) model the water exchange for two coastal areas on a geological timescale (6500 bc and 9000 ad). The model shows a typical progression where the water turnover decreases until a sub-basin is isolated from the sea leading to a reduced dispersal and dilution of potential releases of radionuclides. The transport and residence times for particles in the sea are further explored by Corell and Döös (2013). They model particle-transport with high-resolution advective particle trajectories and explore their behavior as a function of the different shapes (geometries) of coastal areas. In Erichsen et al. (2013), the knowledge of retention and hydrodynamics is combined with an understanding of the accumulation processes in ecosystems. Thus, by combining the measurable and predictable geometry with process understanding of hydrodynamics, particle retention, and ecosystem accumulation, a wide-ranging understanding of the coastal area can be achieved (see Fig. 3).

For the terrestrial environment, water transport is also dependent on the changes in geometric shapes, for example formations of lakes and emergence of watersheds. Lindborg et al. (2013) present the additional process of sedimentation and in-growth of mires in lakes, which is dependent upon, but also affects, the geometric shapes. These geometric changes of the landscape are used by Berglund et al. (2013a) to analyze hydrological conditions from the present to the year 10 000 ad. The transport of groundwater from the repository to the surface is further analyzed by Berglund et al. (2013b) which shows that discharge locations are also coupled to the geometry. To a large extent, the topography determines lakes and wetlands as central recipients of potential releases of radionuclides from an underground repository.

The landscape development modeling (Lindborg et al. 2013) shows how the soil properties are either inherited from the marine phase or modified by resuspension and sedimentation during the shore line displacement process and by mire growth and lake sedimentation. Thus, a mosaic of different future soil properties is created as a consequence of the gradual shore line displacement. These different soil properties affect the sorption (*K*_D_) of radionuclides in different soils. From the studies of *K*_D_ of soils and soil depths by Sohlenius et al. (2013) and Nordén et al. (2010), radionuclide accumulation and mobility can be rescaled within the future landscape mosaic. This rescaling of measured retention factors is further supported by mechanistic understanding implemented in reactive-transport models by Piqué et al. (2013).

The article by Lindborg et al. (2013) describes future landscape development over the next 100 000 years. It shows that the landscape and ecosystems boundaries are affected by a variety of drivers such as permafrost conditions, peat formation, sedimentation, human land use, and shoreline displacement driving ecosystem succession from sea to land. This synthesis of the detailed understanding of processes and how the state of a landscape changes over time is based on the knowledge presented in several of the articles in the special issue. An amazingly understandable future emerges when the different processes and events are compiled. This understanding of landscape development has been the driver of estimations of radionuclide concentrations in the environment and radiation doses to humans (Avila et al. 2013; Saetre et al. 2013).

## Impact Resulting from the Repository on Future Humans and Ecosystems

The final outcome in the assessment is to present the risk for humans and environment of the proposed facility in the future. This takes into account climate change, which induces landscape development that affects the hydrology, hydrodynamics, and ecosystem transfers of matter.

**Fig. 5:**
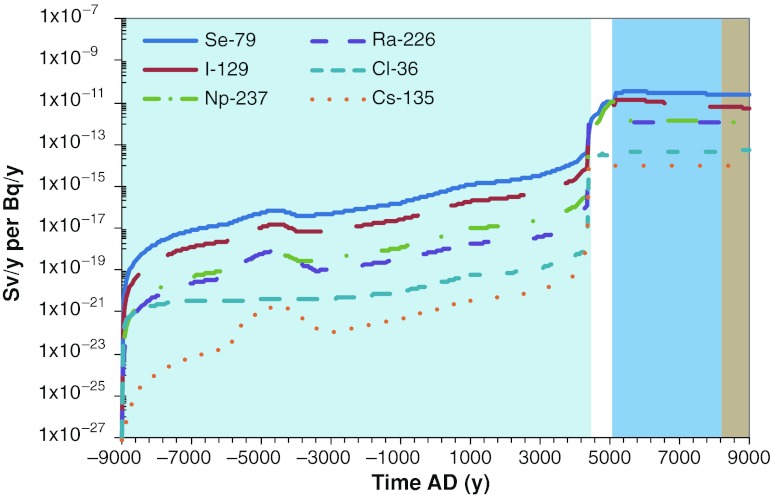
The development of landscape dose conversion factors (LDFs) for a number of dose-contributing radionuclides during an interglacial for one biosphere object (lake/basin no. 116, cf. Lindborg 2010). The object goes through four development stages: (1) the sea stage (*light blue*), (2) the transitional stage (*white*), (3) the lake stage (*blue*), (4) and the terrestrial stage (*brown*)

Avila et al. (2013) show that the model of the long-term transport and accumulation of radionuclides is able to demonstrate that the planned repository can meet the safety criteria. The article discusses the challenges as to how releases in the far future can be handled. Such releases would occur over long periods during which environmental conditions will be continuously varying, due to climate change and ecosystem succession. This is illustrated with resulting “landscape dose conversion factors” (LDFs) in Fig. 5, which shows how the dose, normalized with respect to activity released from the repository, varies over a period of 20 000 years.

Assumptions regarding human behavior affect the final risks. The dose calculation shows that the major pathway for human exposure is through intake of food. Saetre et al. (2013) study the land use and food intake of future inhabitants by selecting a representative person, that is, an individual receiving a dose that is representative of the more highly exposed individuals in the population, by considering the physical and biological characteristics of the potentially most contaminated area and human requirements for energy and nutrients. The intake rate is calculated based on land-use scenarios drawn from self-sustained communities spanning prehistoric times to an industrial age agrarian culture. It shows that consumption of both natural and agricultural contaminated food items are at least five times higher in the historic land-use scenarios, as compared to upper limits based on recent food statistics.

Torudd and Saetre (2013) examined exposure to plants and animals and show that no long-term radiological impact from the repository can be detected. Radiation dose rates to a broad spectrum of relevant organisms were calculated based on data from sampled organisms and simulated activity concentrations. All calculated dose rates for biota were below the default screening dose-rate value of 10 μGy h^−1^ used in the ERICA Tool for integrated assessment (Brown et al. 2008) and also below the lowest band of “derived consideration levels” proposed by the International Commission on Radiological Protection (ICRP 2008).

Disturbances from a repository other than a potential leakage of radionuclides can be addressed using the tools and techniques described herein, as shown by Werner et al. (2013). Here, a novel stepwise methodology is described to assess ecohydrological responses to groundwater diversion from, for example, water-drained pits, shafts, tunnels, and caverns in rock below the groundwater table. For the Forsmark case, artificial water supply to some of the wetlands is suggested to reduce potential negative consequences for rare and protected species, such as the pool frog and fen orchid.

## Conclusions

The wealth of data emerging from the investigations at the candidate sites and the resulting understanding of the biosphere at the sites has facilitated a new approach for assessment and future development of the biosphere. A systematic, scientific, and coherent methodology utilizes the understanding of the spatial and temporal dynamics of the site today as an analog for future conditions.

The articles in this special issue show in different ways how future conditions can be inferred from some few measureable variables. The shore line displacement and the geometry of the landscape are the major variables, which in turn are dependent on the climate reconstruction and modeling. These changes of geometric shape determine the hydrodynamics in the sea and hydrology on land, which are important for the transport and dispersion of radionuclides. The emerging landscape mosaic obtains retention, ecosystem characteristics, and potential land uses from changes in the geometry due to shore line displacement. These properties are derived from measurements (e.g., *K*_D_ values and primary production), which are scaled to the mosaic, or they are modeled with mechanistic models to estimate, e.g., retention, water turnover, and sediment ingrowth. Thus, from robust measurements, simple assumptions, and modeling, very specific results can be obtained defining the future potential areas and populations that will be exposed to the highest risks.

We hope that these examples will stimulate a fruitful discussion as to how environmental threats can be handled with structured methods based on science, and that this work will prove beneficial in applications across a wide range of environmental contexts.
